# VISH-Pred: an ensemble of fine-tuned ESM models for protein toxicity prediction

**DOI:** 10.1093/bib/bbae270

**Published:** 2024-06-06

**Authors:** Raghvendra Mall, Ankita Singh, Chirag N Patel, Gregory Guirimand, Filippo Castiglione

**Affiliations:** Biotechnology Research Center, Technology Innovation Institute, P.O. Box 9639, Abu Dhabi, United Arab Emirates; Biotechnology Research Center, Technology Innovation Institute, P.O. Box 9639, Abu Dhabi, United Arab Emirates; Biotechnology Research Center, Technology Innovation Institute, P.O. Box 9639, Abu Dhabi, United Arab Emirates; Biotechnology Research Center, Technology Innovation Institute, P.O. Box 9639, Abu Dhabi, United Arab Emirates; Graduate School of Science, Technology and Innovation, Kobe University, 1-1 Rokkodai-cho, Nada-ku, Kobe, 657-8501, Japan; Biotechnology Research Center, Technology Innovation Institute, P.O. Box 9639, Abu Dhabi, United Arab Emirates; Institute for Applied Computing, National Research Council of Italy, Via dei Taurini, 19, 00185, Rome, Italy

**Keywords:** peptide toxicity, protein toxicity, deep learning, ESM2 models, fine-tuning, ensemble method

## Abstract

Peptide- and protein-based therapeutics are becoming a promising treatment regimen for myriad diseases. Toxicity of proteins is the primary hurdle for protein-based therapies. Thus, there is an urgent need for accurate *in silico* methods for determining toxic proteins to filter the pool of potential candidates. At the same time, it is imperative to precisely identify non-toxic proteins to expand the possibilities for protein-based biologics. To address this challenge, we proposed an ensemble framework, called VISH-Pred, comprising models built by fine-tuning ESM2 transformer models on a large, experimentally validated, curated dataset of protein and peptide toxicities. The primary steps in the VISH-Pred framework are to efficiently estimate protein toxicities taking just the protein sequence as input, employing an under sampling technique to handle the humongous class-imbalance in the data and learning representations from fine-tuned ESM2 protein language models which are then fed to machine learning techniques such as Lightgbm and XGBoost. The VISH-Pred framework is able to correctly identify both peptides/proteins with potential toxicity and non-toxic proteins, achieving a Matthews correlation coefficient of 0.737, 0.716 and 0.322 and F1-score of 0.759, 0.696 and 0.713 on three non-redundant blind tests, respectively, outperforming other methods by over $10\%$ on these quality metrics. Moreover, VISH-Pred achieved the best accuracy and area under receiver operating curve scores on these independent test sets, highlighting the robustness and generalization capability of the framework. By making VISH-Pred available as an easy-to-use web server, we expect it to serve as a valuable asset for future endeavors aimed at discerning the toxicity of peptides and enabling efficient protein-based therapeutics.

## Introduction

Proteins are natural biological molecules that are essential for wide variety of fundamental cellular processes and molecular functions, such as enzyme activity [1], energy source [2] or activation of the programmed cell death cascade [3, 4]. However, aberrant protein activity can lead to various diseases, including cancer, diabetes and neurological disorders [5–8]. Because of this, the use of proteins or peptides as therapeutic agents is a promising avenue to fight against myriad diseases. Owing to their cost efficiency, high target specificity and biological activity, they are preferred therapeutics over drugs and antibodies [9]. Moreover, to date, over 80 peptides and proteins have been approved globally for usage in the clinic [10]. Nevertheless, a significant obstacle in the development of protein/peptide-based therapeutics is the potential for adverse effects.

**Figure 1:**
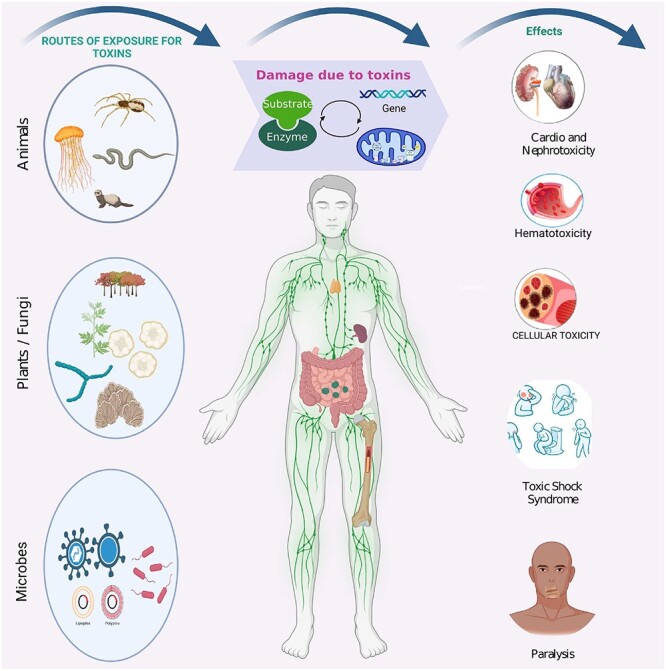
Graphical representation of sources of toxic peptides/proteins in nature and their effects on human health. Figure 1 was created with BioRender.com

Protein toxicity can have deleterious effects on living organisms. Toxic proteins exist naturally in the plant kingdom as well and are present in abundance in animals such as spiders, snakes, scorpions, jellyfish, etc., and myriad microbes such as bacteria and fungi, enabling their enhanced pathogenecity [11]. In addition, there are synthetic toxins, such as carcinogens and mutagens, with high lethality [12]. Toxic proteins from certain bacteria, such as *Listeria monocytogenes*, *Vibrio cholerae*, *Citrobacter freundii*, can cause deadly diseases such as listeriosis, cholera and meningitis, respectively. The mechanisms of action of the different toxins vary, with snake venom inducing ‘neurotoxicity’ causing neuromusclar damage and paralysis or ‘haemotoxicity’ leading to blood coagulation and cardiac arrest [13, 14], whereas scorpion venom can overstimulate neuronal signaling pathways leading to paralysis [15], and jellyfish venom induces anaphylaxis [16]. Fig. 1 illustrates the impact of toxins from various exposure routes.

Although the conventional approach to determine the toxicity of peptides/proteins through experimental evaluation using toxicity assays [17] is the most reliable, it is often laborious and time-consuming. For this reason, with the advent of novel and powerful computational technologies, it is valuable to design accurate *in silico* methods for estimating the toxicity of proteins to advance the development of protein-based therapeutics.

The computational methods that have been proposed to date to predict protein toxicity can be broadly classified into three categories: (a) template-based methods, (b) feature-based methods and (c) deep learning-based methods. Template-based techniques utilize protein sequence alignment tools, such as BLAST [18], to measure the sequence similarity between a target protein and a database of template protein sequences whose toxicity is known and to infer the toxicity of the target protein through *homology* [19, 20]. However, the predictive capacity of these methods hinges on the quality of the template sequence, which is not always available, thereby preventing their widespread application.

Feature-based toxicity predictors such as ClanTox [21], ToxClassifier [22], NNTox [23], ToxinPred [24], ToxinPred2 [25] and ToxinPred3 [26] use a two-step approach. In the first phase, a feature vector representation is devised for individual proteins. This feature vector can represent various properties of the protein, including compositional, evolutionary and physico-chemical features. The compositional features are derived using amino acid compositions such as mono-, di- and tri-petide frequencies as used in BCrystal [27], ProtSol [28] or feature modules from tools such as Pfeature [29] and iFeature [30]. Evolutionary features provide more information than the protein primary sequence [31] and are usually estimated as a position-specific scoring matrix profile, as detailed in [18, 32]. Finally, a variety of numeric physicochemical characteristics such as Cruciani properties, FASGAI vectors, Kidera factors, Aliphatic Index, hydrophobicity, molecular weight, etc., can be extracted using the *peptides* package (v0.3.1) in Python (v3.7.12) [33]. In the second phase, the extracted features are passed to machine learning models such as support vector machines (SVMs) [34, 35], gradient boosting machine (GBM) [36, 37] and feed-forward neural networks [38].

For example, ToxClassifier generates features for protein representations and then uses traditional machine learning models to identify venom toxins [22]. Similarly, popular tools such as ToxinPred [24] and ToxinPred2 [25] use a comprehensive set of characteristics for peptides in combination with machine learning models such as SVMs and random forests [39] to predict the toxicity of peptides, regardless of the source of the peptide (i.e. animal, plant or bacteria). However, the performance of these methods relies heavily on the extracted set of features, necessitating prior expert knowledge of peptides and proteins to ensure the accuracy of predictions.

With the availability of large-scale biological data of public databases such as Uniprot [40], several data-driven deep learning methods have been designed for protein toxicity prediction [41–45]. While Toxify[41] employed a gated recurrent neural network architecture to identify toxic proteins, ATSE [43] adopted graph neural networks with attention mechanisms [46, 47] to learn efficient and discriminative representations from the evolutionary and structural properties of proteins for toxicity prediction. The authors of ATSE [43] further improved their model using information bottlenecks and a transfer learning framework to devise an enhanced toxicity predictor, namely ToxIBTL [44].

Recently, a transformer-based [48] deep learning technique named CSM-Toxin [45] was developed. CSM-Toxin predicts protein toxicity relying solely on the protein primary sequence and creating the largest curated dataset for toxicity prediction. CSM-Toxin is based on ProteinBERT [49], a deep learning model which follows the same principle as the natural language processing model BERT (Bidirectional Encoder Representations from Transformers) [50]. By treating each amino acid as a word and protein sequences as sentences and using attention mechanisms, BERT can capture complex correlations between even distant residues. While fine-tuning ProteinBERT for protein toxicity prediction, the CSM-Toxin model uses the Global Ontology representation of the protein sequence, i.e. concatenating the outputs of each transformer layer to generate an 8493-dimensional binary vector. One of the drawbacks of CSM-Toxin is that the optimal threshold for identifying toxic proteins is 0.968 due to the drastic class imbalance ($\approx $ 1:91) in toxic versus non-toxic [45]. This suggests that for a protein to be classified as toxic the predicted probability score for that protein should be $> 0.968$, whereas in binary classification the predictions range between [0,1]. Hence, all proteins with toxicity scores $\le 0.968$ are classified as negative by CSM-Toxin, reflecting the huge class imbalance in the training data.

In this work, we build an ensemble of fine-tuned deep learning models using ESM2 [51] as the base model. ESM2 is a state-of-the-art transformer-based protein language model trained on $\approx $ 65 million unique protein sequences [51]. ESM2 has been shown to outperform all tested single-sequence protein language models across a range of structure prediction tasks, enabling atomic resolution structure prediction. While the ESM2 model has been benchmarked for structure prediction, it has not been gauged for protein property prediction and has been shown to not scale for protein function prediction [52]. Moreover, the ESM2 models are available with different architectural configurations, that is, with an increasing number of transformer layers leading to increasing number of model parameters.

Our primary contributions are:

- use the largest dataset for toxicity prediction [45] and handle the humongous class-imbalance by devising a smart under-sampling technique (optimal prediction cutoff = 0.5);
- benchmark different ESM2 model configurations for the task of protein toxicity prediction by fine-tuning and providing insights into size versus performance of the models;
- fine-tuning by updating the weights of all transformer layers of ESM2 model in an end-to-end deep learning framework achieves performance similar to state-of-the-art models;
- utilize a feature-based fine-tuning approach on optimal ESM2 model to generate embedding representation for each protein sequence and pass to state-of-the-art models for tabular data, namely LightGBM [53] and XGBoost [54];
- perform a comprehensive comparison with latest protein toxicity prediction models across myriad quality metrics [accuracy, precision, recall, F1, Matthews correlation coefficient (MCC) etc.] which handle the class imbalance on multiple independent datasets;
- assemble the top-performing fine-tuned models to achieve over 10% improvements in critical quality metrics such as MCC [55] and F1-score [56, 57], given the large class-imbalance in the data;
- provide the VISH-Pred framework as a web-server at http://ec2-35-170-123-194.compute-1.amazonaws.com:7860/ for ease of access of non-experts.

**Figure 2:**
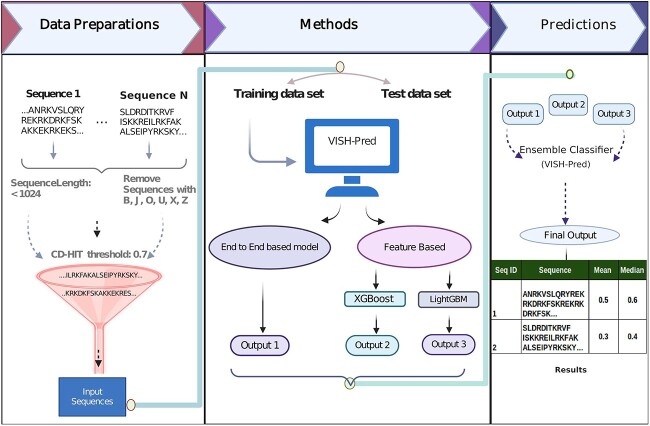
Representative diagram of the VISH-Pred framework for protein toxicity prediction.

Figure 2 shows a flowchart of the proposed VISH-Pred framework for protein toxicity prediction.

## Materials and methods

### Data collection and curation

#### Data preparation

We followed the protocol used by CSM-Toxin [45] to assemble the largest dataset for the task of peptide toxicity prediction. Specifically, the data were collected from UniProt release 2022_04 [58]. Previously reviewed proteins using the query ‘(keyword: KW-0800) AND (reviewed:true)’ were classified as toxic (i.e. positive samples). Those reviewed using the query ‘NOT (keyword:KW-0800) NOT (keyword: KW-0200) AND (reviewed:true)’ were classified as non-toxic and non-allergic proteins (i.e. negative samples). This resulted in a total of 567 390 proteins including 7543 positive and 559 847 negative samples, respectively.

Protein sequences containing nonstandard residues, such as B, J, O, U, X and Z, were discarded, thus reducing the positive set to 7398 and the negative set to 557 354 protein samples, respectively. CD-HIT version 4.8.1 [59] was used with a similarity threshold of 0.7 [60] to remove redundant proteins sets and make the data set unbiased. Thus, proteins with high sequence similarity i.e. point mutations and small truncations were removed and only representative samples were kept to create an unbiased training set. The final real data set covered 2309 positive and 212 541 negative protein samples, respectively. Additionally, the minimum size of a peptide in our dataset is 11 amino acids (AA) and the median size of protein is 302 AAs and the mean protein size was 341 AAs. A distribution of the protein size included in our dataset is depicted as a histogram in Supp. Figure S1.

#### Data curation

In CSM-Toxin [45], the following steps were undertaken to create an independent test set, a blind test set from the real dataset and the remaining real dataset used for training:

- a small set of 204 toxic and 2337 non-toxic proteins collected after July 2022 and with low sequence similarity to training set was considered as an independent test set;
- a subset comprising 236 positive and 21 294 negative proteins was extracted from the real dataset as a blind set;
- the remaining real dataset (2073 positive and 191 247 negative samples) was considered as a training set and equally split in five parts for cross-validation in [45].

Since the ESM2 model can reliably perform inference only for proteins of size less than 1024 amino acids, we took some additional steps including the following:

- we removed protein sequences of length $l> 1024$ to have a revised independent test set consisting of 202 toxic and 2160 non-toxic proteins;
- the revised blind set comprised 222 positive and 20 329 negative samples after removing large protein sequences;
- the reduced real dataset had 2015 toxic and 182 561 non-toxic samples after removing proteins with $l> 1024$.
- we implemented a simple under-sampling technique to reduce the enormous class imbalance ratio ($\approx 1:91$). Keeping the set of positive samples fixed, we divided the set of negative samples into 10 random groups and fine-tuned ESM2 models for each set comprising 2015 positive samples and 18 256 negative samples, resulting in 10 different models.
- each revised real dataset was stratified into two-parts comprising the training set (80%) and the validation set (20%) while maintaining the class-imbalance;
- each model had an optimal classification cutoff of 0.5 and a reduced class-imbalance ratio ($\approx 1:9$) and this setup enabled easy ensemble of fine-tuned ESM2 models.

**Figure 3:**
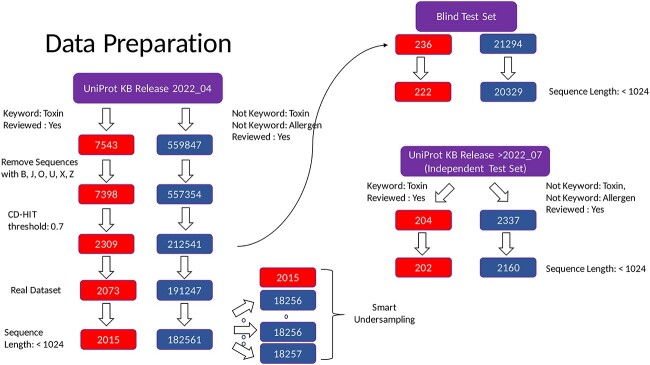
Data preprocessing pipeline. Data were collected from UniProt KB using a keyword search for toxic and non-toxic proteins. Sequences with non-canonical amino acids and high similarity were discarded using CD-HIT. All proteins of length $l>$ 1024 amino acids were removed from the curated datasets. Finally, a smart under-sampling approach was utilized to reduce the class-imbalance and divide the real dataset into 10 subsets.

The data preparation protocol is detailed in Fig. 3. The preprocessing steps used on the training data are consistent with those used for processing the test sets.

#### Independent bacterial dataset

An independent dataset comprising peptides with antibacterial activity as well as toxic bacterial peptides was obtained from [61]. This dataset comprised 2170 peptides, all below the sequence similarity threshold of 0.7 with real dataset using CD-HIT and obtained from the public source named Database of Antimicrobial Activity and Structure of Peptides (DBAASP) [62]. Each peptide sequence varied in length between 4 and 119 AAs and were classified into two groups, i.e. the non-toxic class with high antibacterial activity and low cytotoxicity and the toxic class with low antibacterial activity and high cytotoxicity. The cutoff values to annotate a peptide as toxic was minimum inhibitory concentration $\le 14.97 \mu $M, or cytotoxic concentration at $50\%$ ($\text{CC}_{50}$) $\ge 60.91 \mu $M, or hemolytic concentration at $50\%$ ($\text{HC}_{50}$) $\ge 105.7 \mu $M. There were a total of 1071 non-toxic peptides and 1099 toxic peptides in this independent bacterial dataset.

### Methods overview

Protein toxicity prediction can be computationally modeled as a classification task. We learn a mapping function $\mathbf{g}$ that takes as input a protein representation, say $x_{p}$, and outputs the predicted toxicity score $\hat{y}_{p}= \mathbf{g}(x_{p};\theta )$, where $\hat{y}_{p} \in [0,1]$. If $\mathbf{L}$ is the model-specific loss function, then the classification task reduces to estimating the parameters $\theta $ which minimizes $\text{min}_{\theta } \sum _{p} \mathbf{L}(y_{p},\hat{y}_{p})$, where $y_{p}$ is the ground truth label and takes the value 0 (for non-toxic proteins) or 1 (for toxic proteins).

In this article, the mapping function $\mathbf{g}$ is based on the ESM2 model [51]. ESM2 is a transformer-based protein language model trained on $\approx $ 65 million unique protein sequences using a masked language modeling objective [63]. The ESM2 model is trained to predict the identity of randomly selected amino acids in a protein sequence by observing their context w.r.t. rest of the sequence. This enables the model to learn dependencies between the amino acids. While the training objective of the ESM2 model is relatively simple and unsupervised, by performing well on this task over millions of diverse proteins, the model can internalize sequence patterns across evolution. As a result, the model can efficiently capture structure as it can be linked to the sequence patterns. In addition, the ESM2 models were trained with different configurations as depicted in Supplementary Table S1. Increasing the size of the models lead to large improvements in quality of protein structure [51].

In our work, we fine-tune the ESM2 model transformer with classification head [51] for the binary classification, i.e. toxic versus non-toxic, based on the sequence. Since the dataset used for fine-tuning the ESM2 models are highly imbalanced, we used the weighted binary cross-entropy loss as the loss function ($\mathbf{l}$) which can formally described as follows: 


(1) \begin{align*}& \mathbf{L}_{w\text{BCE}} = \frac{1}{N_{p}}\sum_{p} - w^{1}_{p} y^{1}_{p} \log\left( \mathbf{g}(x_{p};\theta) \right) - w^{0}_{p}(1-y^{0}_{p}) \log\left( 1-\mathbf{g}(x_{p};\theta) \right),\end{align*}

where $N_{p}$ is the number of proteins, $w^{1}_{p}$ and $w^{0}_{p}$ are the weights for the classes 1 and 0 and are chosen to represent the imbalance in the dataset, i.e. $w^{1}_{p}=0.9$ and $w^{0}_{p}=0.1$. $\mathbf{g}(x_{p};\theta )$ is the probability of class 1 and $(1-\mathbf{g}(x_{p};\theta ))$ is the probability of class 0. By minimizing the loss function $\mathbf{L}$, we can fine-tune the models to accurately estimate the toxicity of proteins.

#### Fine-tuning ESM2 models

**Figure 4:**
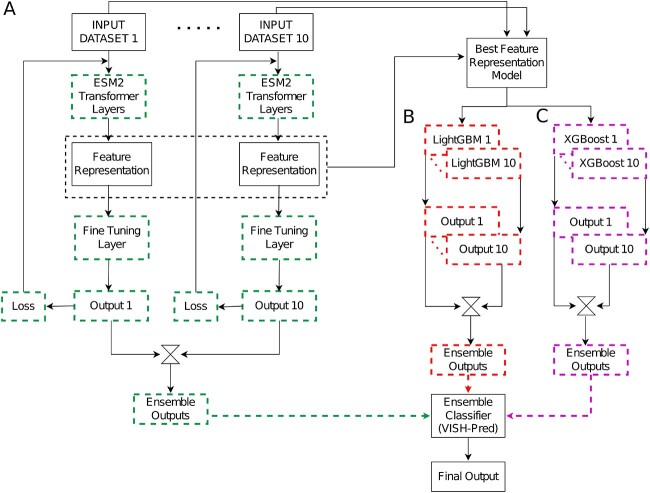
VISH-Pred model architecture. (A) 10 ESM2 models handle the humongous class imbalance and are fine-tuned for the protein toxicity prediction task. All the parameters for the boxes highlighted in ‘green’ are fine-tuned. (B) The fine-tuned ESM2 transformer model with the best performance in the validation set generated the local feature representations for each of the 10 parts of the reduced real data set. Each part (1 out of 10) is utilized to build an optimal LightGBM classifier via a 5-fold cross-validation. (C) The same local feature representations for each of the revised real dataset is used to build an optimal XGBoost classifier (through 5-fold cross-validation). The outputs of the 10 fine-tuned models, the 10 LightGBM classifiers and the 10 XGBoost classifiers are passed to the ensemble classifier (VISH-Pred) to have the final output score $(\hat{y}_{p}$) for a given protein $p$.

We downloaded and fine-tuned the ESM2 models of varying size from the HuggingFace library [64] in Python and followed a protocol. Since we divided our reduced real dataset into 10 parts, we fine-tuned one ESM2 model for each part while keeping configuration fixed (i.e. defined number of transformer layers and learnable parameters). The output of ESM2 model (i.e. the last transformer layer) is a local representation of the protein sequence and is connected with a trainable classification head comprising feed forward layers followed by a softmax layer to categorize proteins into toxic or non-toxic classes. This is shown in Fig. 4A. Each ESM2 transformer model, along with the classification head, is fine-tuned in the corresponding training set and evaluated in the validation set using the Adam optimizer [65].

The Adam optimizer depends on several parameters including learning rate, batch size, maximum number of epochs and early stopping criterion as described in [57, 66]. We fine-tuned each model for a maximum of 10 epochs with a learning rate of 1e-4 and weight decay of 0.01. A low learning rate and weight decay are required to prevent the appearance of large absolute values of the gradients that can significantly change the pretrained model weights. We used a single Nvidia RTX A6000 GPU with 48GB RAM for the fine-tuning process. We therefore used only those ESM2 models that could be loaded on a single GPU for fine-tuning. In addition, as the size of the ESM2 model increases, the number of samples that can be passed to the model in a given batch decreases. To have a fair comparison between ESM2 models of varying configurations, we limited the batch size to 4 and introduced a gradient accumulation step [64]. Gradient accumulation is a way to virtually increase the batch size during the fine-tuning process and is useful when the available GPU memory is insufficient to accommodate the desired batch size. In gradient accumulation, gradients are computed for smaller batches and accumulated (summed or averaged) over multiple iterations instead of updating the model weights after every batch. We fixed the gradient accumulation step to 4, thus virtually increasing the batch size to 16. Additionally, we set the early stopping criterion to 5, i.e. if the model performance on the validation set does not improve for 5 consecutive epochs, then we stop training the model further to avoid over-fitting. As our dataset is heavily imbalanced, we implemented our own weighted loss trainer to inform the model to give more weights to the toxic proteins (class 1).

Once we fine-tuned each ESM2 transformer model along with the classification head (1 out of 10 parts), we concatenated the outputs of the 10 models for the ensemble classifier. We also identified the ESM2 transformer model $+$ classifier (1 out of 10) with the best performance on validation set as depicted in Fig. 4A. The output feature representations from the corresponding fine-tuned ESM2 transformer model are similarly used by other state-of-the-art machine learning classifiers, such as LightGBM and XGBoost. Fig. 4A highlights the fine-tuning process of the ESM2 transformer model along with the classification head on each revised real dataset. Their generated outputs are concatenated and used in the ensemble classifier.

#### Traditional machine learning models

It has been previously shown that nonlinear ML techniques such as XGBoost and LightGBM can be used efficiently for a variety of bioinformatics problems [6, 27, 28, 37, 56, 67–69]. Here we too used such state-of-the-art nonlinear ML methods for tabular data, XGBoost [27, 54] and LightGBM [53], as mapping function $\mathbf{g}$. Both XGBoost and LightGBM are based on GBMs [36]. GBM belongs to a family of predictive methods that uses an iterative strategy, that is, the learning framework will consecutively fit new models to have an accurate estimate of the response variable after each iteration. The advantage of the boosting procedure is that it works on decreasing the bias in the model, without increasing the variance. A more scalable and accurate version of GBM is XGBoost [54] and light-weight version of GBM is LightGBM [53]. These methods use a scalable end-to-end tree-boosting system with a weighted quantile sketch for approximate tree learning. Both XGBoost and LightGBM can scale for a large number of samples using very little computational resources.

Here, the input to these models are the output embeddings from the last transformer layer of the best-performing fine-tuned ESM2 model resulting in the feature representation vector, $\hat{x}_{p}$, for the protein $p$. Thus, our predicted toxicity for a given protein $p$ can be formally written as $\hat{y}_{p}= \mathbf{g}(\hat{x}_{p};\theta )$. We used the ‘xgboost’ package (v1.7.6) and ‘lightgbm’ package (v4.0.0) available in Python for building XGBoost and LightGBM models. For each revised real dataset, we build one optimal XGBoost (see Fig. 4C) and one optimal LightGBM model (see Fig. 4B), respectively, resulting in 10 optimal models for the 10 parts. We perform hyperparameter optimization using 5-fold stratified cross-validation. To do cross-validation, we shuffled the rows of each revised real dataset and then randomly split the data into five parts, using a combination of four parts as training set and one part as validation set (while maintaining the class-imbalance) to identify the optimal set of hyperparameters. This process is repeated five times and the hyperparameters with best average performance are then selected as optimal hyperparameters. These hyperparameters are used to build the final model on the entire revised real dataset.

#### VISH-Pred: ensemble classifier

Ensemble methods use multiple learning algorithms to obtain better predictive performance than any individual component [70] for a given supervised task. For instance, in [71], the authors demonstrated that, for identifying drugs that can be repurposed against COVID-19, an ensemble of the results from different methodologies can provide better performance than individual models. In a similar vein, we take a consensus, i.e. the average ($\mu $) of the predicted toxicity scores from the top-performing fine-tuned ESM2 transformer model with classification head, XGBoost and LightGBM models on independent test sets as the output of the ensemble classifier ($\hat{y}_{p}$). We claim that since our models are based on different configurations (ESM2 model) and learning algorithms (tree-based versus end-to-end deep learning), it is essential to take an ensemble of the top predictive models to attain optimal performance as illustrated in Table 1. Supp. Figure S2 highlights detailed architecture of the proposed VISH-Pred framework.

#### Evaluation metrics

Following [56, 57], the performance of proposed VISH-Pred ensemble classifier was compared with various other *in silico* protein toxicity predictors using quality metrics such as accuracy, area under receiver operating curve (AUC), and MCC. We assessed several other evaluation metrics, based on TP, TN, false positives (FP) and false negative (FN). TP represents the set of proteins which are toxic (true label is 1) and are correctly identified by a given method as toxic, i.e. $\hat{y}_{p} \ge 0.5$. Similarly, TN represents the set of proteins which are non-toxic (true label is 0) and are correctly identified by a given method as non-toxic ($\hat{y}_{p} < 0.5$). Based on the same principle, the score distribution for the FP set represents the score distribution for all proteins whose true label is 0 but are incorrectly identified as toxic. The score distribution for the FN set represents the score distribution for all proteins whose true label is 1 but are incorrectly identified as non-toxic. The metrics used for evaluation include 


\begin{gather*} \text{Accuracy (ACC)} = \frac{TP+TN}{TP+FP+TN+FN} \\ \text{MCC} = \frac{TP \times TN - FP \times FN}{\sqrt{(TP+FP) \times (TP+FN) \times (TN+FP) \times (TN + FN)}} \\ \text{Recall (Rec)} = \frac{TP}{TP+FN} \\ \text{Precision (Prec)} = \frac{TP}{TP+FP} \end{gather*}

\begin{gather*} \text{F1-score (F1)} = \frac{2 \times \text{Prec} \times \text{Rec}}{\text{Prec} + \text{Rec}} \end{gather*}

Finally, each of previous toxicity predictor was downloaded from their respective github repository (except CSM-Toxin) and was utilized in an inference phase to predict toxicity for test proteins/peptides with default parameter settings. For CSM-Toxin, the protein sequences were passed to their web-server as a FASTA file and the quality metrics were applied on the obtained results accordingly.

#### Docking protocol for case study

The goal of the molecular docking investigation was to find a common ligand (small molecule) with high binding affinity against two hazardous peptides from *Escherichia coli* and *Klebsiella pneumoniae*, which were correctly identified by the VISH-Pred model as toxic proteins.

Due to the unavailability of the 3D structures of these target peptides, homology modeling of target sequences was performed using AlphaFold [72]. Although AlphaFold uses deep learning to predict protein structures, it does so within the context of homology modeling. Traditionally, homology modeling methods such as SwissModel [73] and ModWeb [74] involve comparing similar protein structures to predict the structure of a target protein. AlphaFold makes this prediction by utilizing evolutionary connections relevant to the protein sequence, similar to such standard homology modeling approaches, using a cutting-edge deep learning model. AlphaFold model provides high predicted local Distance Difference Test (plDDT) scores for the target structures [75]. High plDDT scores (e.g. > 80) indicate high confidence in the structure of the residue, and low plDDT scores (e.g. < 50) indicate that the residues are in intrinsically disordered protein regions [72]. Supp. Figures S3a and S3b highlight the target structures along with their corresponding plDDT scores. To restore equilibrium in the modeled structures, a molecular dynamics simulations was run for 100 ns.

The ChEMBL database was selected for the molecular docking study [76] comprising $\approx $ 1000 compounds with known antibacterial activity [77]. The compounds chosen from the ChEMBL database include a diverse range of molecules, including those that have been shown to bind to toxic proteins, as well as medications that target disorders caused by toxic proteins/peptides. We have ensured that the selection criteria are consistent with our research goals of identifying possible interactions between relevant chemicals and biological targets. Finally, molecular docking was employed using the Maestro module of Schrödinger software (Release 2023-2) using pre-defined parameters for protein and compound preparation.

Next, the homology modeled structures were then processed through the Schrödinger’s Glide (grid-based ligand docking with extra precision) protein preparation wizard, which included pre-processing steps such as adding hydrogens, removing water and optimizations carried out using a refinement technique that aids the OPLS 2005 force field [78, 79]. Furthermore, the Ligprep suite of the Maestro module was utilized for compound minimization (we applied a flexible docking approach because of the small size of the peptides to obtain the docking score and inter-molecule interactions). Finally, the binding pocket dimension were set with $30 \overset{\circ }{\text A} \times 30 \overset{\circ }{\text A} \times 30 \overset{\circ }{\text A}$ grid box size and the docking experiment was performed with the Schrödinger molecular modeling package’s Glide program.

## Experimental results

### Benchmarking on validation sets

We benchmarked the performance of the fine-tuned ESM2 transformer models with classification head and different configurations (in terms of transformer layers and parameter size) as reported in Supp. Table S2. From Supp. Table S2, it can be seen that in the validation sets for the 10 parts of the reduced real dataset, the performance of the ESM2 models increased as the size of the model increased. The mean $\pm $ standard deviations of the F1 and MCC scores for ESM2: T6-12M, ESM2: T12-35M, ESM2: T30-150M and ESM2: T33-650M models were 0.882 $\pm $ 0.015, 0.894 $\pm $ 0.011, 0.908 $\pm $ 0.014, 0.909 $\pm $ 0.012 and 0.869 $\pm $ 0.017, 0.882 $\pm $ 0.027, 0.898 $\pm $ 0.013, 0.899 $\pm $ 0.022, respectively, in the validation sets as observed in Supp. Table S2 and Supp. Figures S4a–d.

However, we observed that feature-based models such as XGBoost (XGB) and LightGBM (LGBM) models built on top of features generated by best fine-tuned ESM2 model (of a particular configuration) had better performance w.r.t. all evaluation metrics as illustrated in Supp. Figures S4e–l. The LGBM (ESM2: T6-8M), LGBM (ESM2: T12-35M), LGBM (ESM2: T30-150M) and LGBM (ESM2: T33-650M) models achieved F1 scores of 0.942 $\pm $ 0.015, 0.945 $\pm $ 0.013, 0.947 $\pm $ 0.009, 0.952 $\pm $ 0.009 and MCC scores of 0.936 $\pm $ 0.017, 0.939 $\pm $ 0.014, 0.942 $\pm $ 0.010, 0.947 $\pm $ 0.010, respectively, on the validation sets as depicted in Supp. Table S2 and Figures S4e–h. Similarly, XGB (ESM2: T6-8M), XGB (ESM2: T12-35M), XGB (ESM2: T30-150M) and XGB (ESM2: T33-650M) models attained F1 scores of 0.942 $\pm $ 0.015, 0.943 $\pm $ 0.014, 0.948 $\pm $ 0.010, 0.954 $\pm $ 0.008 and MCC scores of 0.936 $\pm $ 0.017, 0.937 $\pm $ 0.015, 0.943 $\pm $ 0.011, 0.949 $\pm $ 0.009, respectively, on the validation sets as highlighted in Supp. Table S2 and Figures S4i–l. We observed from Supp. Table S2 that XGBoost and LightGBM models could handle class imbalance better since these models directly optimized the F1 scores during cross-validation compared with the fine-tuned ESM2 transformer models with classification head which optimized the weighted binary cross-entropy loss ($\mathbf{l}_{w\text{BCE}}$). Finally, the ESM2: T30-150M and ESM2: T33-650M along with their feature-based counterparts, XGB (ESM2: T30-150M), XGB (ESM2: T33-650M), LGBM (T30-150M) and LGBM (T33-650M) had better performance than the small sized ESM2 models (see Supp. Table 2). Hence, we took the ensemble of these models to build our final ensemble classifier, VISH-Pred.

We also benchmarked the run time complexity of fine-tuning the ESM2 models as indicated in Supp. Table S3 and Supp. Figure S5a. All the ESM2 models ran up to the maximum setting of 10 epochs as illustrated in Supp. Figure S5b. The run time increased quadratically as the size of the ESM2 model increased from 8 million parameters ($\mu (t) = 4016.5$ s) to 650 million parameters ($\mu (t)=16\,664$ s). Here $\mu (t)$ represents the mean run time in seconds, i.e. the average run time for fine-tuning each ESM2 model on a revised real dataset. Additionally, the training loss decreased with the number of epochs, while the validation loss decreased initially and increased with the number of epochs, as highlighted in Supp. Figure S5b. However, the validation MCC which is used as the early stopping criterion was maximum around the $9{\text{th}}$ or $10{\text{th}}$ epoch for most of the fine-tuned ESM2 models and as a result majority of the models ran up to the maximum 10 epochs as depicted in Supp Figure S5b.

### Performance on small test set

**Figure 5:**
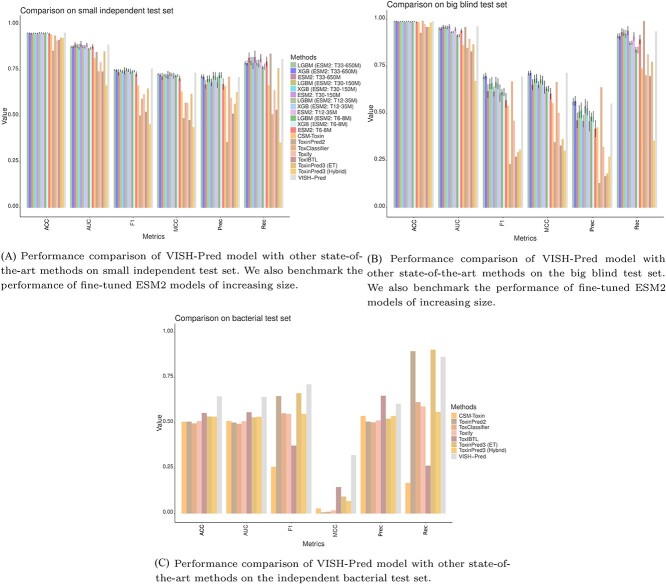
Comprehensive comparison and benchmarking of the fine-tuned ESM2 models and the ensemble classifier (VISH-Pred) against state-of-the-art methods such as Toxify, ToxClassifier, ToxIBTL, CSM-Toxin, ToxinPred2, ToxinPred3 on three independent test sets. The models are evaluated using various evaluation metrics, in particular, metrics such as MCC and F1-score to handle the class imbalance in the test sets.

We compared the predictive performance of state-of-the-art toxicity predictors such as ToxClassifier, ToxITBL, Toxify, ToxinPred2, ToxinPred3 (ET), ToxinPred3 (Hybrid) with fine-tuned ESM2 models and our ensemble classifier, VISH-Pred, across different quality metrics on the small test set as depicted in Table 1 and Fig. 5(A). The class-imbalance ratio in this dataset was $\approx $ 1:10. We observed that the VISH-Pred model achieved the best performance with respect to metrics (w.r.t.) such as F1 (**0.759**), ACC (**0.956**) and MCC (**0.737**) and second best performance for AUC (**0.891**) as illustrated in Table 1. The performance of VISH-Pred model was superior than other state-of-the-art toxicity predictors with over $10\%$ improvement w.r.t. F1 and MCC scores over the next best peptide toxicity predictor (CSM-Toxin).

**Table 1:** Comparison of performance of fine-tuned ESM2 models, ensemble classifier (VISH-Pred) with other state-of-the-art *in silico* peptide toxicity predictors on the small independent test set. Here ‘${+}$’ represents the best model and ‘${*}$’ represents the second best model

**Method**	**F1**	**AUC**	**ACC**	**MCC**	**Prec**	**Rec**
CSM-Toxin	0.667	0.818	0.943	0.635	0.665	0.668
ESM2: T6-8M	0.73${\pm }$0.0127	0.881${\pm }$0.0109	0.95${\pm }$0.00382	0.706${\pm }$0.0133	0.675${\pm }$0.0298	0.798${\pm }$.0262
ESM2: T12-35M	0.744${\pm }$0.014	0.89${\pm }$0.00937	0.952${\pm }$0.00377	0.721${\pm }$0.0146	0.684${\pm }$0.0278	0.816${\pm }$0.0218
ESM2: T30-150M	0.747${\pm }$0.0181	**0.894${\pm }$0.0156**	0.952${\pm }$0.00327	0.725${\pm }$0.0203	0.684${\pm }$0.0189	**0.824${\pm }$0.0323** $^{*}$
ESM2: T33-650M	0.739${\pm }$0.0163	**0.891${\pm }$0.0127** $^{*}$	0.951${\pm }$0.00351	0.717${\pm }$0.018	0.673${\pm }$0.0222	0.82${\pm }$0.0268
LGBM (ESM2: T6-8M)	0.744${\pm }$0.00513	0.869${\pm }$0.00305	0.955${\pm }$0.00157	0.72${\pm }$0.00551	0.723${\pm }$0.0145	0.766${\pm }$0.0081
LGBM (ESM2: T12-35M)	0.754${\pm }$0.00796	0.881${\pm }$0.00593	**0.956${\pm }$0.00228** $^{+}$	0.731${\pm }$0.00826	0.721${\pm }$0.0194	0.791${\pm }$0.0143
LGBM (ESM2: T30-150M)	0.749${\pm }$0.00479	0.885${\pm }$0.00929	0.954${\pm }$0.00164	0.726${\pm }$0.00521	0.703${\pm }$0.0165	0.801${\pm }$0.0218
LGBM (ESM2: T33-650M)	0.752${\pm }$0.00348	0.88${\pm }$0.0035	0.955${\pm }$0.000932	0.729${\pm }$0.00376	0.718${\pm }$0.00896	0.79${\pm }$0.00817
ToxClassifier	0.595	0.745	0.941	0.574	0.715	0.51
ToxIBTL	0.524	0.744	0.917	0.479	0.514	0.535
Toxify	0.62	0.794	0.913	0.571	0.599	0.641
ToxinPred2	0.504	0.851	0.858	0.49	0.359	**0.842** $^{+}$
ToxinPred3 (ET)	0.651	0.854	0.93	0.621	0.568	0.762
ToxinPred3 (Hybrid)	0.456	0.668	0.927	0.44	0.632	0.356
XGB (ESM2: T6-8M)	0.746${\pm }$0.00516	0.871${\pm }$0.00689	0.955${\pm }$0.00142	0.722${\pm }$0.00561	**0.724${\pm }$0.0148** $^{+}$	0.77${\pm }$0.016
XGB (ESM2: T12-35M)	0.75${\pm }$0.00732	0.879${\pm }$0.00842	0.955${\pm }$0.00222	0.727${\pm }$0.00759	0.718${\pm }$0.0207	0.787${\pm }$0.0201
XGB (ESM2: T30-150M)	0.744${\pm }$0.00753	0.879${\pm }$0.0144	0.953${\pm }$0.00158	0.72${\pm }$0.00915	0.704${\pm }$0.0175	0.79${\pm }$0.0325
XGB (ESM2: T33-650M)	0.75${\pm }$0.00596	0.88${\pm }$0.00604	0.955${\pm }$0.0014	0.726${\pm }$0.00657	0.714${\pm }$0.0126	0.79${\pm }$0.0136
VISH-Pred	**0.759** $^{+}$	**0.891** $^{*}$	**0.956** $^{+}$	**0.737** $^{+}$	0.713	0.812

The fine-tuned ESM2 models (ESM2: T30-150M and ESM2: T33-650M) achieved the best AUC (**0.894**) and second best AUC (**0.891**), respectively, while the feature-based LGBM (ESM2: T12-35M) achieved the joint best accuracy (**0.956**) along with the VISH-Pred model as highlighted in Fig. 5(A). Similarly, the ESM2: T30-150M achieved the second best recall score (**0.824**) following ToxinPred2 (**0.842**) and the XGB (ESM2: T6-8M) achieved the best precision score (**0.724**) on the small test set. The majority of the fine-tuned ESM2 models already attained performance better than state-of-the-art methods such as CSM-Toxin, ToxinPred2 and ToxinPred3 for several quality metrics.

### Performance on blind test set

We compared the performance of proposed methods with other protein toxicity predictors on the big blind test set where the class imbalance ratio was $\approx $ 1:91. As a result, the predictive performance of all methods decreased, in particular, for the F1 and MCC quality metrics, which handle the class-imbalance in the data as depicted in Table 2. VISH-Pred model accomplished the best scores for MCC (**0.716**), AUC (**0.991**) metrics and second best performance for F1 (**0.696**) and ACC (**0.991**) metrics as showcased in Fig. 5(B) and Table 2. Interestingly, LGBM (ESM2: T33-650M) and XGB (ESM2: T33-650M) models procured optimal performance w.r.t. F1, ACC, MCC and precision metrics. Furthermore, the ToxClassifier model achieved the best accuracy (**0.993**) and best precision (**0.639**) at the expense of much lower recall (0.703) compared with the fine-tuned ESM2 models. Similarly, ToxinPred2 secured the best recall (**0.991**) at the expense of extremely low precision (0.132). Although the VISH-Pred model cannot outperform individual peptide toxicity predictors on precision and recall, it achieved an increase of over $5\%$ in performance for relevant quality metrics (F1 and MCC) over the next best model (ToxClassifier) given the humongous class-imbalance in the blind test set.

**Table 2:** Comparison of performance of fine-tuned ESM2 models, ensemble classifier (VISH-Pred) with other state-of-the-art *in silico* peptide toxicity predictors on the big blind test set. Here ‘${+}$’ represents the best model and ‘${*}$’ represents the second best model

**Method**	**F1**	**AUC**	**ACC**	**MCC**	**Prec**	**Rec**
CSM-Toxin	0.54	0.864	0.986	0.555	0.426	0.739
ESM2: T6-8M	0.57${\pm }$0.0379	0.94${\pm }$0.0107	0.985${\pm }$0.00256	0.606${\pm }$0.0284	0.42${\pm }$0.0433	0.894${\pm }$0.0232
ESM2: T12-35M	0.608${\pm }$0.0441	0.949${\pm }$0.00961	0.987${\pm }$0.00272	0.64${\pm }$0.0332	0.46${\pm }$0.0531	0.909${\pm }$0.0214
ESM2: T30-150M	0.617${\pm }$0.0251	**0.964${\pm }$0.0105** $^{+}$	0.987${\pm }$0.00155	0.652${\pm }$0.0178	0.46${\pm }$0.0323	**0.94${\pm }$0.0224** $^{*}$
ESM2: T33-650M	0.619${\pm }$0.0351	**0.961${\pm }$0.0092** $^{*}$	0.987${\pm }$0.00205	0.653${\pm }$0.0274	0.464${\pm }$0.0401	0.933${\pm }$0.0194
LGBM (ESM2: T6-8M)	0.613${\pm }$0.0153	0.914${\pm }$0.00621	0.989${\pm }$0.000953	0.632${\pm }$0.0108	0.483${\pm }$0.0214	0.839${\pm }$0.0134
LGBM (ESM2: T12-35M)	0.666${\pm }$0.0274	0.933${\pm }$0.00366	0.99${\pm }$0.00139	0.682${\pm }$0.0215	0.539${\pm }$0.0359	0.874${\pm }$0.00861
LGBM (ESM2: T30-150M)	0.657${\pm }$0.0269	0.959${\pm }$0.00755	0.989${\pm }$0.00155	0.683${\pm }$0.0198	0.51${\pm }$0.0334	0.929${\pm }$0.0164
LGBM (ESM2: T33-650M)	**0.696${\pm }$0.013** $^{*}$	0.952${\pm }$0.00569	**0.991${\pm }$0.000611** $^{*}$	0.713${\pm }$0.0102	0.563${\pm }$0.0188	0.912${\pm }$0.0119
ToxClassifier	0.67	0.849	**0.993** $^{+}$	0.667	**0.639** $^{+}$	0.703
ToxIBTL	0.271	0.83	0.96	0.33	0.168	0.698
Toxify	0.462	0.897	0.976	0.504	0.323	0.815
ToxinPred2	0.232	0.96	0.929	0.348	0.132	**0.991** $^{+}$
ToxinPred3 (ET)	0.295	0.868	0.96	0.364	0.182	0.775
ToxinPred3 (Hybrid)	0.308	0.673	0.983	0.302	0.271	0.356
XGB (ESM2: T6-8M)	0.613${\pm }$0.0207	0.914${\pm }$0.00758	0.989${\pm }$0.00133	0.631${\pm }$0.0146	0.484${\pm }$0.0284	0.837${\pm }$0.0166
XGB (ESM2: T12-35M)	0.658${\pm }$0.028	0.934${\pm }$0.004	0.99${\pm }$0.00145	0.676${\pm }$0.022	0.528${\pm }$0.0364	0.877${\pm }$0.0093
XGB (ESM2: T30-150M)	0.661${\pm }$0.0317	0.957${\pm }$0.00851	0.99${\pm }$0.0019	0.686${\pm }$0.0226	0.517${\pm }$0.0402	0.923${\pm }$0.019
XGB (ESM2: T33-650M)	**0.699${\pm }$0.0165** $^{+}$	0.952${\pm }$0.00754	**0.991${\pm }$0.000805** $^{*}$	**0.715${\pm }$0.0124** $^{*}$	**0.567${\pm }$0.0248** $^{*}$	0.911${\pm }$0.0159
VISH-Pred	**0.696** $^{*}$	**0.964** $^{+}$	**0.991** $^{*}$	**0.716** $^{+}$	0.553	0.937

### Performance on bacterial test set

Finally, we compared our proposed framework with other state-of-the-art protein toxicity predictors in the independent bacterial test set. The bacterial test set was balanced in terms of number of toxic and non-toxic bacterial peptides. However, the sequence similarity with our training set was low and the lengths of bacterial peptides were $l <$ 120 amino acids. This differs significantly from the length distribution of the reduced real dataset utilized for fine-tuning the ESM2 models as highlighted in Supp. Figure S1. This resulted in a significant drop in performance of all methods for various quality metrics including accuracy (ACC), AUC and MCC.

The VISH-Pred model still achieved the best performance w.r.t. different quality metrics including F1 (**0.713**), AUC (**0.644**), ACC (**0.647**), MCC (**0.322**) and second best performance for precision (0.606). ToxITBL attained the best precision (**0.65**) at the expense of very low recall (**0.264**) and ToxinPred3 (ET) had the best recall (**0.905**) but relatively low precision (**0.524**). In line with our previous observation, the VISH-Pred model achieved over $10\%$ performance for F1 and MCC quality metrics than the next best predictor, i.e. ToxinPred3 (ET) and ToxITBL respectively. Additionally, on this difficult independent test set, VISH-Pred impressively accomplished over $10\%$ gain in performance for AUC and ACC quality metrics than the competing protein toxicity predictors.

### Case study on bacterial test set

We undertook a case study of the two most toxic peptides from the bacterial test set and were accurately identified by the VISH-Pred model, one from *Escherichia coli* and one from *Klebsiella pneumoniae*, respectively, as indicated in Supp. Table S4. We wanted to identify whether a known small molecule could bind and inhibit the toxic peptides. To answer this, we performed a docking study to identify a common ligand with high binding affinity against both toxic peptides.

We first showed that the 3D structures of the peptides had more than $70\%$ predicted local Distance Difference Test (plDDT) scores via AlphaFold, as highlighted in Supp. Figures S2a and S2b. The plDDT scores can take values in the range of $[0, 100]$. High plDDT scores (e.g. $\ge 70$) indicate high confidence in the residue structure, and low plDDT scores (e.g. $\le 50$) indicate lower confidence in the estimated structure. The homology modeled structures were docked with CheMBL compounds and top-10 hits were identified based on docking scores (see Supp. Tables S5 and S6).

**Figure 6:**
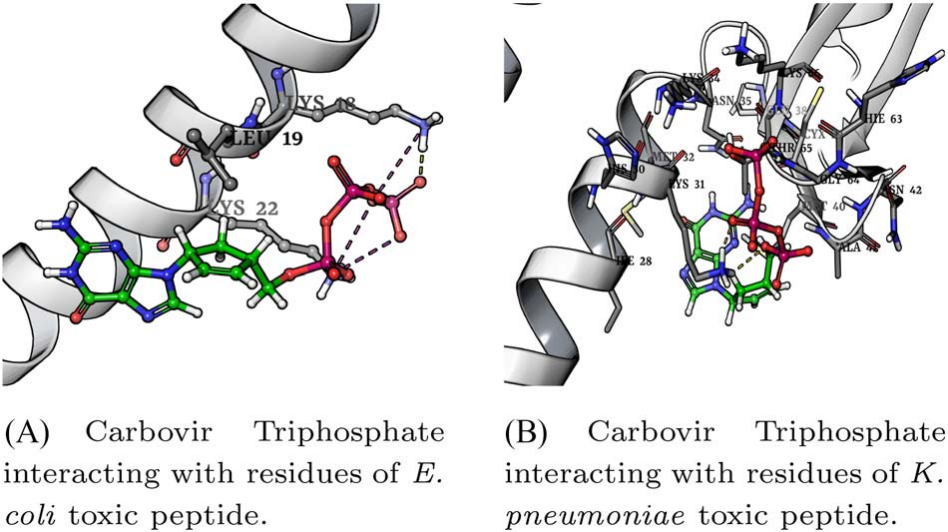
Carbovir Triphosphate binding with residues of toxic peptides from *E. coli* and *K. pneumoniae*, respectively.

Among the top-hits, Carbovir Triphosphate was pinpointed as a potential binder (inhibitor) with both the peptides. The docking of Carbovir Triphosphate and *E. coli* peptide formed one hydrogen bond (Lys 18) and two salt bridges (Lys 18 and Lys 22) with the binding energy of -5.33 Kcal / mol as shown in Fig. 6(A). Similarly, the peptide obtained from *Klebsiella pneumoniae* and Carbovir Triphosphate docked with a binding energy of -5.78 Kcal/mol forming four hydrogen bonds (Lys 31, Asn 35 and Thr 65) as illustrated in Fig. 6(B). Based on both docking incidents (see Supp. Tables S5 and S6), we can conclude that the same compound can potentially act as an inhibitor to target highly toxic peptides from two different model organisms.

Here we recognize that binding affinity is one of the steps in antidote preparation. A good antidotal action is dependent on factors beyond binding affinity. Although docking studies can give significant insight into possible binding interactions, the development of an effective antidote requires a thorough examination across several parameters. These include binding selectivity, functional effects on the target molecule, pharmacokinetic characteristics, safety profile, dose–response relationship, delivery method and clinical validation via preclinical and clinical trials. As a result, while our docking experiments give information on possible interactions between small molecules and hazardous peptides or proteins, the eventual evaluation of these compounds into effective antidotes requires extensive research across numerous domains to ensure therapeutic efficacy and safety.

Finally, we provide a small subset of 36 bacterial proteins as an example file on our web-server and it takes approximately $10$ min for the VISH-Pred framework to provide the results.

## Discussion

A significant obstacle in protein or peptide therapeutics is the hazard of protein toxicity. It is imperative to evaluate the toxicity of proteins where conventional experimental techniques are expensive and time-consuming. Hence, precise *in silico* models that predict the toxicity of proteins with high efficacy and high performance are highly desirable and the motivation behind the proposed VISH-Pred framework. The word ‘VISH’ in Sanskrit refers to venom/poison, and hence we named our framework as VISH-Pred.

Using the largest curated dataset of 2015 toxic and 182 561 non-toxic proteins [45], we performed a comprehensive comparison of various classes of *in silico* protein toxicity predictors including feature-based methods such as ToxClassifier, ToxinPred2 and ToxinPred3 as well as end-to-end deep learning approaches such as Toxify, ToxIBTL and CSM-Toxin. We observed low predictive performance for these methods particularly on quality metrics such as F1-score and MCC on two independent datasets, a small test set comprising 202 toxic and 2160 non-toxic proteins and a bigger test set consisting of 222 toxic and 20 329 non-toxic proteins, respectively. This was indicative of the poor generalization and handling of class imbalance by the prior predictive tools.

Employing the ESM2 model as the base transformer model [51] and the simplest representation of protein i.e. amino acid sequence as input, we designed VISH-Pred, a novel framework for protein toxicity estimation. By just taking the protein sequence as input and no additional computationally expensive post-processing step, such as BLAST or motif scan as implemented by ToxinPred2 and ToxinPred3, VISH-Pred is an efficient high-throughput protein toxicity screening tool. We fine-tuned ESM2 transformer models of various architectural configurations (see Supp. Tables S1 and S2 and Supp. Figure S4) and identified the optimal fine-tuned ESM2 model for the task of predicting protein toxicity. The output embedding representation of these models was then passed to tree-based machine learning methods such as LightGBM [53] and XGboost [54] classifiers. By taking an ensemble of fine-tuned ESM2 models and corresponding feature-based XGBoost and LightGBM models, we create the final VISH-Pred framework. The VISH-Pred framework was able to accurately and robustly identify both toxic and non-toxic proteins, outperforming a priori approaches by at least 10$\%$ across three independent test sets for different quality metrics (specifically the F1 and MCC quality metrics).

An important step undertaken in the VISH-Pred framework was to handle the humongous class imbalance in the reduced real dataset. By fixing the toxic proteins as one part and dividing the non-toxic proteins into 10 parts, we reduced the class-imbalance ratio from 1:91 to 1:9, allowing the usage of the cutoff value of 0.5 as the optimal threshold for the protein toxicity classification. Furthermore, by implementing custom weighted trainer for the weighted loss function (i.e. $\mathbf{l}_{w\text{BCE}}$) in the ESM2 models with classification head and providing the inverted imbalance ratio as the weights to ‘scale_pos_weight’ parameter in the LightGBM and XGBoost models, we observed that these models already achieved performance better than the state-of-the-art for F1, AUC, ACC and MCC metrics as depicted in Tables 1 and 2. Additionally, the XGBoost and LightGBM models achieved better F1 and MCC scores than the fine-tuned ESM2 models (see Tables 1 and 2) as they directly optimized for the F1-score during cross-validation.

By outperforming other models on F1 and MCC metrics (see Tables 1, 2 and 3), the VISH-Pred model can not only accurately identify toxic proteins but has fewer falsely classified toxic proteins (FP) and fewer incorrectly classified non-toxic proteins (FN) simultaneously. This is different from other classifiers such as ToxClassifier and ToxIBTL (see Tables 2 and 3) which can accurately expand the pool of non-toxic proteins (high precision) but at the expense of misclassifying several toxic proteins as non-toxic (FN) proteins. This can be hazardous when designing protein-based therapeutics. Similarly, methods such as ToxinPred2 and ToxinPred3 (see Tables 1, 2 and 3) focus on not missing out on any toxic protein (high recall) but at the expense of misclassifying several non-toxic candidates as toxic. This is also detrimental for the protein-based biologic development as potentially feasible protein candidates rue being missed. The advantage of the proposed VISH-Pred framework is that it attains both high precision and high recall simultaneously as depicted from our results.

**Table 3:** Comparison of performance of VISH-Pred with other state-of-the-art *in silico* peptide toxicity predictors on the bacterial test set. Here ‘${+}$’ represents the best model and ‘${*}$’ represents the second best model

**Method**	**F1**	**AUC**	**ACC**	**MCC**	**Prec**	**Rec**
CSM-Toxin	0.258	0.51	0.506	0.0284	0.539	0.169
ToxClassifier	0.553	0.495	0.497	0.00983	0.503	0.615
ToxIBTL	0.375	0.559	0.555	0.146	**0.65** $^{+}$	0.264
Toxify	0.55	0.509	0.51	0.0175	0.514	0.591
ToxinPred2	0.648	0.502	0.507	0.00592	0.507	0.897
ToxinPred3 (ET)	0.664	0.531	0.536	0.094	0.524	**0.905** $^{+}$
ToxinPred3 (Hybrid)	0.55	0.534	0.535	0.0685	0.539	0.561
VISH-Pred	**0.713** $^{+}$	**0.644** $^{+}$	**0.647** $^{+}$	**0.322** $^{+}$	**0.606** $^{*}$	0.866

In a difficult independent bacterial test set comprising predominantly small peptides, most of the previous methods achieved poor predictive performance (MCC < 0.1) as shown in Table 3. VISH-Pred was the only model that achieved a reasonable MCC score (MCC = 0.322) and outperformed other methods by more than $10\%$ for quality metrics such as F1, AUC, ACC and MCC metrics (see Fig. 5C). Additionally through a docking-based study, we illustrated that a common ligand (Carbovir Triphosphate) can render two most toxic peptides, (one from *E. coli* and one from *K. pneumoniae*) identified by the VISH-Pred model from the bacterial test set, to be ineffective.

In conclusion, we propose a novel ensemble framework based on fine-tuned ESM2 protein language models, namely VISH-Pred, for the task of protein toxicity prediction. VISH-Pred framework takes just the primary sequence representation of proteins (peptides) as input and overcomes limitations such as a two-stage classifier with a separate feature selection step and handling of the humongous class imbalance in the data. VISH-Pred significantly outperforms all sequence-based protein toxicity predictors with respect to various evaluation metrics such as ACC, MCC, AUC and F1-score across different independent test sets. To facilitate ease of usage, our model is available through an easy-to-use web interface. We expect that VISH-Pred will serve as a valuable asset for upcoming endeavors aimed at discerning the toxicity of proteins, enabling efficient protein-based therapeutics.

**Key Points**

- Ensemble of fine-tuned ESM2 models (VISH-Pred) outperforms existing state-of-the-art protein toxicity predictors like CSM-Toxin, ToxinPred2, ToxinPred3, ToxClassifier, ToxIBTL, Toxify, etc. on three independent test sets.
- VISH-Pred efficiently handles humongous class-imbalance in training set (ratio of 1:91) for toxic versus non-toxic peptides using an under-sampling approach and ensembles fine-tuned models to mitigate the risk of majority class prediction.
- VISH-Pred achieves over 10% increase in performance for key metrics, F1-score and MCC, which are imperative for screening candidates for protein-based therapeutics.
- Benchmarking different ESM2 language model configurations provided insights into size versus performance trade-offs.
- VISH-Pred is available as a user-friendly web service for the ease of usage of nonexperts.

## Supplementary Material

VISH_Pred_Supplementary_bbae270

## Data Availability

All the data required to train the model are available via https://bitbucket.org/ascherslab/csm-toxin/src/master/data_processing/ (accessed on 17 January 2023) and VISH-Pred model is available as a web-server for testing at: http://ec2-35-170-123-194.compute-1.amazonaws.com:7860/.

## Author Biographies

**Raghvendra Mall** is a Director at Technology Innovation Institute. His expertise lies in machine learning and computational biology. His research focuses on inferring gene regulatory networks, differential network analysis of biological networks and protein property prediction using deep learning.

**Ankita Singh** is a Senior Scientist at the Technology Innovation Institute (TII) with a background in biophysics and computational biology. Her research is focused on developing new computational methods and software tools and biological databases to help answer fundamental biological questions.

**Chirag N. Patel** is a Senior Scientist at the Technology Innovation Institute (TII). His areas of interest include computational biology investigations for biomedical treatments to enhance pharmacological efficacy, including molecular modeling and applied machine learning.

**Gregory Guirimand** is a Lead Researcher at Technology Innovation Institute. His expertise lies in synthetic biology and metabolic engineering of microorganisms for the bioproduction of valuable compounds.

**Filippo Castiglione** is the Executive Director of Bioinformatics at Technology Innovation Institute. He has expertise in computational biology, machine learning, mathematical modeling and complex systems with a focus on the immune system.
