# Supplementary material for: VISH-Pred: an ensemble of fine-tuned ESM models for protein toxicity prediction
Source: Brief Bioinform. 2024 Jun 6;25(4):bbae270. doi: 10.1093/bib/bbae270 (PMC11154842; doi:10.1093/bib/bbae270)
Supplement: VISH_Pred_Supplementary_bbae270 [file vish_pred_supplementary_bbae270.pdf]

VISH-Pred: An ensemble of fine-tuned ESM models for protein toxicity prediction

Raghvendra Mall, Ankita Singh, Chirag N. Patel, Gregory Guirimand, Filippo Castiglione

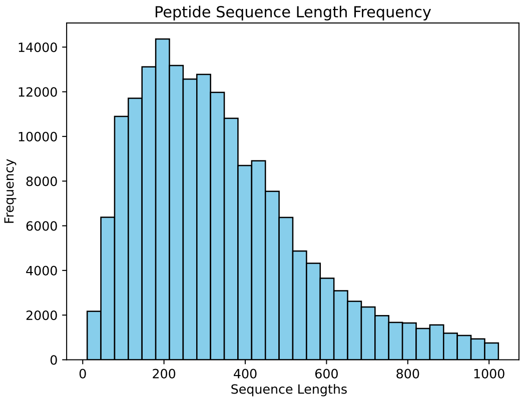

Figure 1: Distribution of length of proteins used for fine-tuning the ESM2 models

**Model Name**	**Number of transformers layers**	**Number of parameters**
ESM2: T48-15B	48	15 Billion
ESM2: T36-3B	36	3 Billion
ESM2: T33-650M	33	650 Million
ESM2: T30-150M	30	150 Million
ESM2: T12-35M	12	35 Million
ESM2: T6-8M	6	8 Million

Table 1: ESM2 transformer models with different configurations

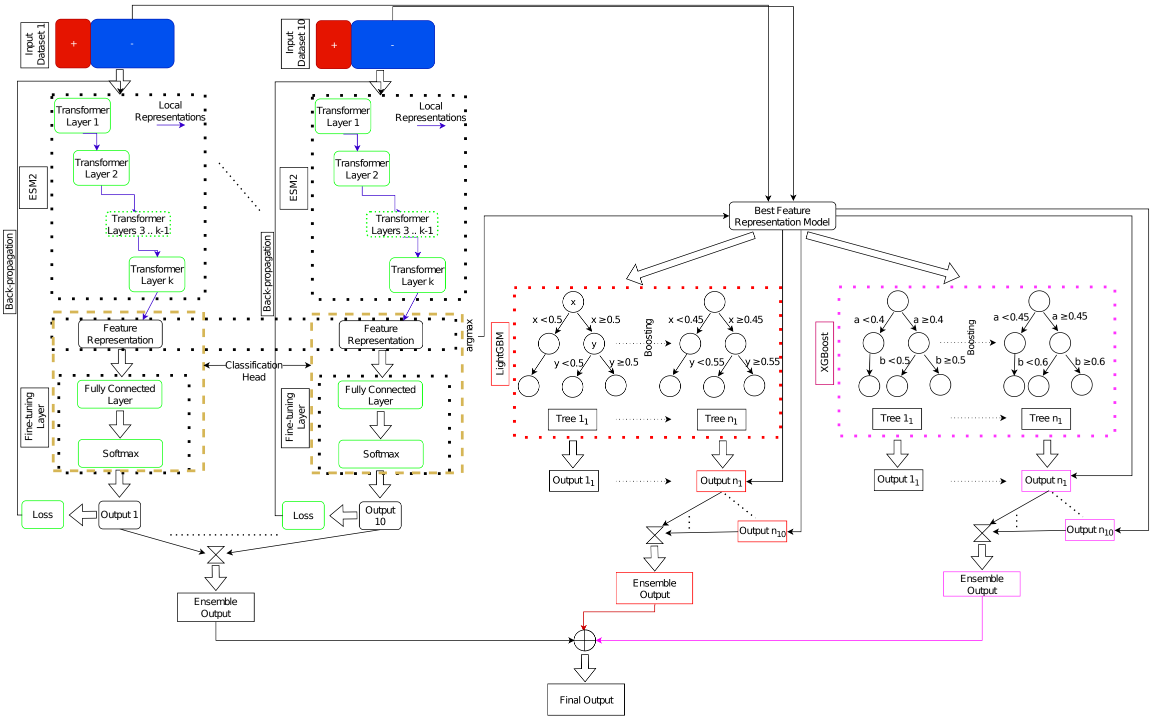

Figure 2: VISH-Pred model architecture. A) 10 ESM2 models along with their classification heads handle the humongous class imbalance and are fine-tuned for the protein toxicity prediction task. All the parameters for the boxes highlighted in 'green' are fine-tuned. The classification head is highlighted in 'gold'. B) The fine-tuned ESM2 transformer model with the best performance on validation set generated the local feature representations for each of the 10 parts of the reduced real dataset. Each part (1 out of 10) is utilized to build an optimal LightGBM classifier via a 5-fold cross-validation. C) The same local feature representations for each of revised real dataset is used to build an optimal XGBoost classifier (through 5-fold cross-validation). The outputs of the 10 fine-tuned models, the 10 LightGBM classifiers and the 10 XGBoost classifiers are ensembled together to have the final output score ($\hat{y}_p$) for a given protein p.

Method	F1	AUC	ACC	MCC	Prec	Rec
ESM2: T6-8M	0.882 +/- 0.0152	0.936 +/- 0.0132	0.976 +/- 0.00309	0.869 +/- 0.0169	0.88 +/- 0.028	0.885 +/- 0.0282
ESM2: T12-35M	0.894 +/- 0.0114	0.941 +/- 0.0109	0.979 +/- 0.00239	0.882 +/- 0.0127	0.896 +/- 0.027	0.893 +/- 0.024
ESM2: T30-150M	0.908 +/- 0.0141	0.953 +/- 0.00814	0.982 +/- 0.00232	0.898 +/- 0.0127	0.899 +/- 0.0127	0.918 +/- 0.0165
ESM2: T33-650M	0.909 +/- 0.012	0.955 +/- 0.0103	0.982 +/- 0.00245	0.899 +/- 0.0134	0.898 +/- 0.0218	0.921 +/- 0.0217
LGBM (ESM2: T6-8M)	0.942 +/- 0.0151	0.977 +/- 0.0124	0.988 +/- 0.00293	0.936 +/- 0.0167	0.921 +/- 0.0122	0.964 +/- 0.0245
LGBM (ESM2: T12-35M)	0.945 +/- 0.0128	0.976 +/- 0.0105	0.989 +/- 0.0025	0.939 +/- 0.0142	0.929 +/- 0.0124	0.961 +/- 0.021
LGBM (ESM2: T30-150M)	0.947 +/- 0.00927	0.984 +/- 0.00677	0.989 +/- 0.00188	0.942 +/- 0.0103	0.920 +/- 0.0117	0.977 +/- 0.0135
LGBM (ESM2: T33-650M)	0.952 +/- 0.00912	0.983 +/- 0.0103	0.99 +/- 0.00182	0.947 +/- 0.0101	0.931 +/- 0.0115	0.974 +/- 0.0161
XGB (ESM2: T6-8M)	0.942 +/- 0.0148	0.978 +/- 0.0113	0.988 +/- 0.00292	0.936 +/- 0.0165	0.921 +/- 0.0133	0.964 +/- 0.0222
XGB (ESM2: T12-35M)	0.943 +/- 0.0136	0.977 +/- 0.0105	0.989 +/- 0.0027	0.937 +/- 0.0151	0.925 +/- 0.0146	0.963 +/- 0.021
XGB (ESM2: T30-150M)	0.948 +/- 0.00968	0.983 +/- 0.00594	0.989 +/- 0.00198	0.943 +/- 0.0108	0.923 +/- 0.0117	0.975 +/- 0.0113
XGB (ESM2 T33-650M)	0.954 +/- 0.00848	0.983 +/- 0.00785	0.991 +/- 0.00165	0.949 +/- 0.00947	0.934 +/- 0.0066	0.974 +/- 0.0158

Table 2: Comparison of performance of fine-tuned ESM2 models of increasing size along with their corresponding feature-based XGBoost and LightGBM models on the validation sets for the 10 parts of the revised real dataset. Here we report mean ± standard deviations in the results.

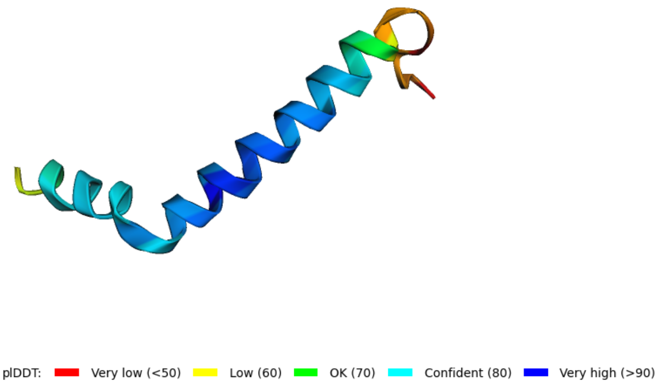

(a) Alphafold predicted optimal structure for the *Escherchia coli* toxic peptide.

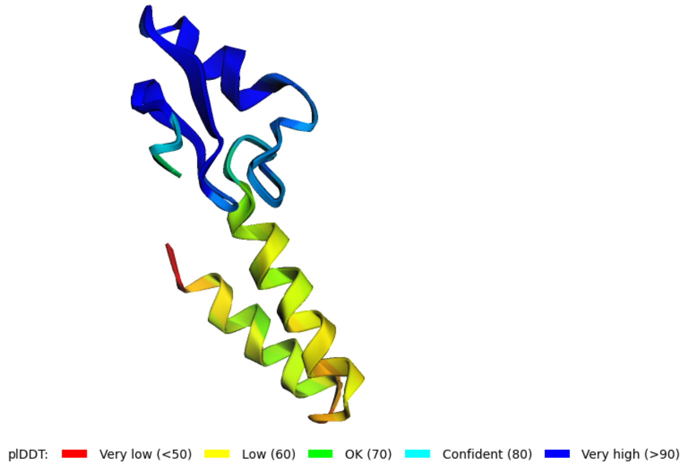

(b) Alphafold predicted optimal structure for the *Klebsiella pneumoniae* toxic peptide.

Figure 3: Predicted structures of toxic peptides have high plDDT scores.

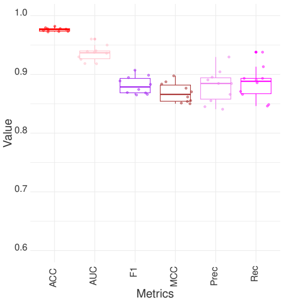

(a) Validation performance of fine-tuned ESM2: T6-8M models.

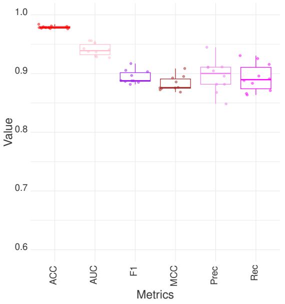

(b) Validation performance of fine-tuned ESM2: T12-35M models.

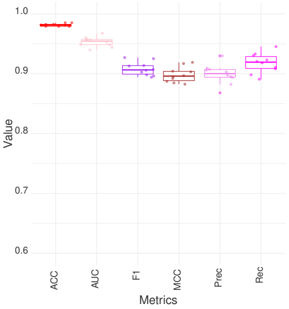

(c) Validation performance of fine-tuned ESM2: T30-150M models.

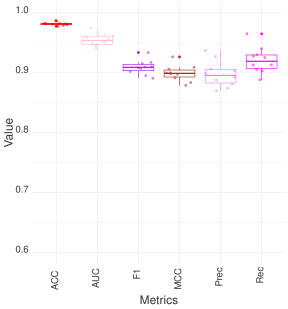

(d) Validation performance of fine-tuned ESM2: T33-650M models.

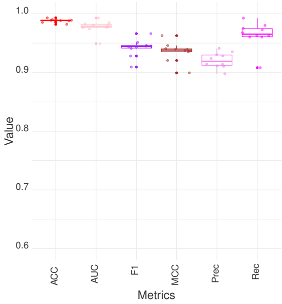

(e) Validation performance of XGBoost models based on optimal ESM2: T6-8M transformer model features.

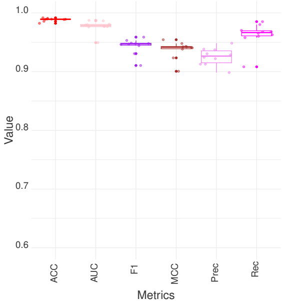

(f) Validation performance of XGBoost models based on optimal ESM2: T12-35M transformer model features.

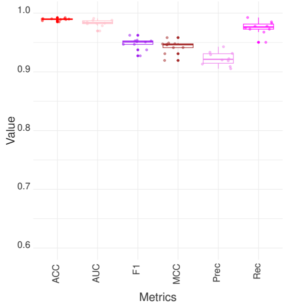

(g) Validation performance of XGBoost models based on optimal ESM2: T30-150M transformer model features.

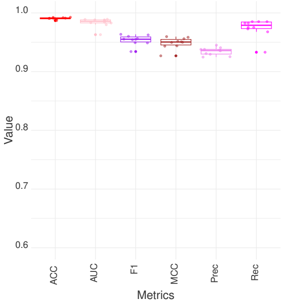

(h) Validation performance of XGBoost models based on optimal ESM2: T33-650M transformer model features.

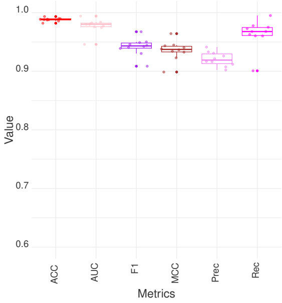

(i) Validation performance of Lightgbm models based on optimal ESM2: T6-8M transformer model features.

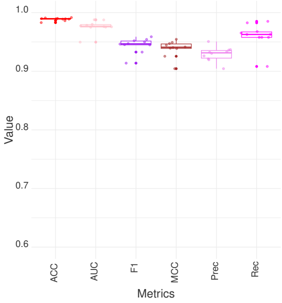

(j) Validation performance of Lightgbm models based on optimal ESM2: T12-35M transformer model features.

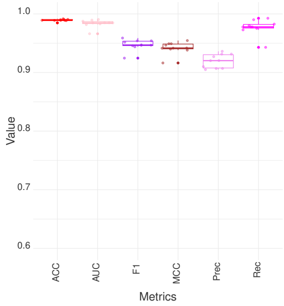

(k) Validation performance of Lightgbm models based on optimal ESM2: T30-150M transformer model features.

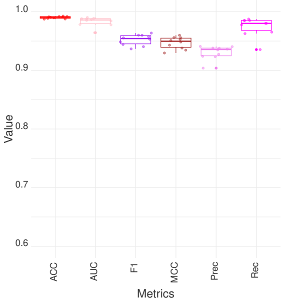

(l) Validation performance of Lightgbm models based on optimal ESM2: T30-150M transformer model features.

Figure 4: Comprehensive benchmarking of the fine-tuned ESM2 models of increasing size along with their corresponding feature-based XGBoost and LightGBM models on the validation sets for the 10 parts of the revised real dataset.

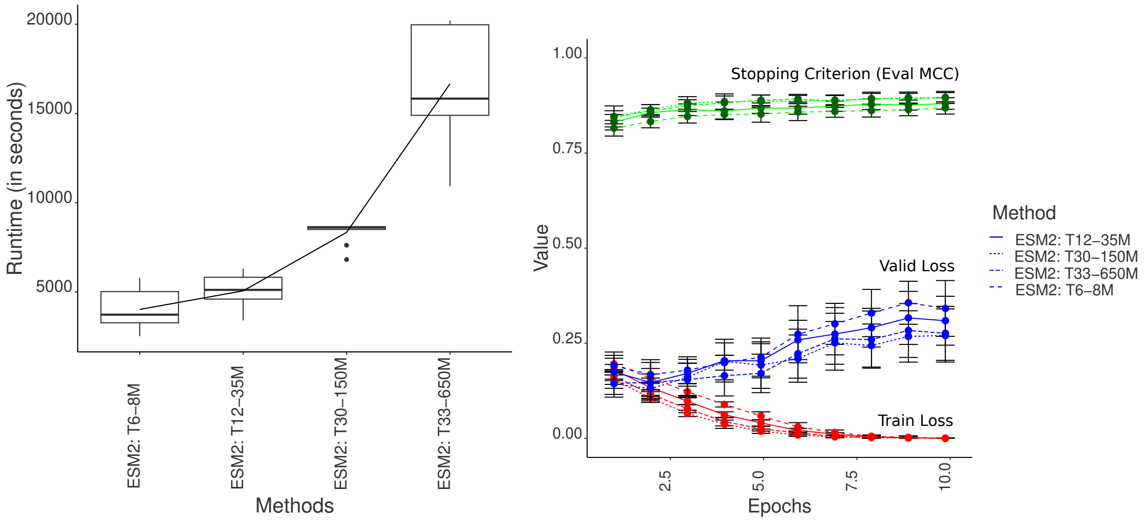

(a) Run time for fine-tuned ESM2 models

(b) Loss for fine-tuned ESM2 models

Figure 5: Run time for the fine-tuning the ESM2 transformer models of increasing parameter configurations and the evolution of loss functions for these models for protein toxicity prediction task.

ESM2: T6-8M	ESM2: T12-35M	ESM2: T30-150M	ESM2: T33-650M
3346.451	6299.984	8650.8849	20148.800
3779.798	5373.515	7615.201	19992.502
2521.900	3990.758	8671.0115	14920.125
3242.564	6122.486	8664.0277	20216.280
4314.153	4611.900	8622.7579	14888.907
5318.852	3406.577	6813.7814	13976.765
5250.398	4592.550	8586.7628	15804.735
5775.700	5969.901	8471.4435	19915.155
2950.373	4991.895	8599.1893	15861.083
3669.245	5234.727	8660.6472	10922.809

Table 3: Run time complexity of the fine-tuned ESM2 models for the 10 revised real datasets.

Sequence	Label	VISH-Pred (mean)	VISH-Pred (median)	Biological Sample	LGBM (mean)	XGB (mean)	ESM2 (mean)
FKLGSFLKKAWKSKL AKKLRAKGKEMLKDY AKGLLEGGSEEVPGQ	1	0.996	0.999	Escherichia coli (ATCC 25922 / KCTC 1682)	0.999	0.999	0.991
GWINEEKIQKKIDERMGNTVLGGMA KAIVHKMAKNEFQCMANMDMLGNCE KHCQTSGEKGYCHGTKCKCGTPLSY	1	0.998	0.999	Klebsiella pneumoniae	0.998	0.999	0.998

Table 4: VISH-Pred results on the two most toxic bacterial peptides.

ChEMBL Id	ChEMBL Name	Docking Score	Glide Score
CHEMBL1162433	ADEFOVIR DIPHOSPHATE	-5.736	-5.82
CHEMBL485652	STAVUDINE TRIPHOSPHATE	-5.543	-5.624
CHEMBL1350	TILUDRONIC ACID	-5.517	-5.586
CHEMBL1159869	GANCICLOVIR TRIPHOSPHATE	-5.401	-5.481
CHEMBL473882	ENTECAVIR TRIPHOSPHATE	-5.365	-5.444
**CHEMBL1201395**	**CARBOVIR TRIPHOSPHATE**	**-5.335**	**-5.415**
CHEMBL1674	TELBIVUDINE 5'TRIPHOSPHATE	-5.291	-5.373
CHEMBL4802226	APRICITABINE TRIPHOSPHATE	-5.144	-5.223
CHEMBL1673	ACYCLOVIR TRIPHOSPHATE	-5.135	-5.215
CHEMBL3544916	GUADECITABINE	-5.101	-5.567

Table 5: Docking score for most toxic peptide identified from *Escherchia coli.*

ChEMBL Id	ChEMBL Name	Docking Score	Glide Score
CHEMBL555230	None	-6.753	-6.753
CHEMBL12318	CLODRONIC ACID	-6.204	-6.211
CHEMBL4594373	CLEVUDINE TRIPHOSPHATE	-5.992	-6.075
CHEMBL871	ETIDRONIC ACID	-5.964	-6.218
CHEMBL476960	VOGLIBOSE	-5.831	-5.837
CHEMBL50300	FOSFONET	-5.803	-5.861
**CHEMBL1201395**	**CARBOVIR TRIPHOSPHATE**	**-5.782**	**-5.862**
CHEMBL3544916	GUADECITABINE	-5.712	-6.178
CHEMBL3544980	GUADECITABINE SODIUM	-5.712	-6.178
CHEMBL473159	PHLOROGLUCINOL	-5.53	-5.551

Table 6: Docking score for the most toxic peptide identified from *Klebsiella pneumoniae.*
